# Determinants of Inappropriate Admissions in County Hospitals in Rural China: A Cross-Sectional Study

**DOI:** 10.3390/ijerph15061050

**Published:** 2018-05-23

**Authors:** Yan Zhang, Liang Zhang, Haomiao Li, Yingchun Chen

**Affiliations:** 1 School of Medicine and Health Management, Tongji Medical College, Huazhong University of Science and Technology, Wuhan 430030, China; yanzhang@hust.edu.cn (Y.Z.); zhangliang@mails.tjmu.edu.cn (L.Z.); lihaomiao@hust.edu.cn (H.L.); 2 Research Centre for Rural Health Service, Key Research Institute of Humanities & Social Sciences of Hubei Provincial Department of Education, Wuhan 430030, China

**Keywords:** New Rural Cooperative Medical System, inappropriate admission, county hospital, appropriateness evaluation protocol, rural China

## Abstract

Inappropriate admissions have contributed to the rapid increase in hospitalisations in rural China. This study characterised the degree and determinants of inappropriate admissions in county hospitals. We used expert consultation to develop an appropriateness evaluation protocol that included nine requirements for services and 21 indicators of disease severity. A total of 2230 medical records from 2014 were collected from five county hospitals by stratified cluster sampling and evaluated for appropriateness using the protocol in 2016. The determinants of inappropriate admissions were analysed by two-level logistic regression. The overall inappropriate admission rate was 15.2%. Patients aged <20 years (19.3%), patients in the paediatrics department (22.9%), patients with lower disease severity (22.3%), and patients without complications (17.0%) were more likely to have been inappropriately admitted than other groups. Age, treating department, disease severity, causes of hospitalisation, complications, and length of stay were determinants of inappropriate admission. Policymakers must act to reduce the high prevalence of inappropriate admissions in county hospitals in rural China, by guiding patients to seek primary care and changing the motivating mechanism of these hospitals.

## 1. Introduction

The term “inappropriate admission” refers to patients receiving unnecessary inpatient care rather than outpatient services [1,2]. It is a major form of excess hospitalisation demand, and results from intentional or unintentional decision-making by both patients and physicians [3]. Inappropriate admission is also a cause of increasing health expenditures and waste [4].

Residents in rural China seek healthcare services from a three-tier (village–town–county) healthcare delivery system, and all inpatient services are supplied by township and county hospitals. A typical rural county has 1–2 county hospital (400–500 staff, 350–500 beds, 400–800 thousand population) and 20–30 township hospitals (20–50 staff, 20–40 beds, 10–30 thousand population). The higher is the level of the institution, the stronger is the service capability, the greater is the distance a patient must travel and the higher is the medical cost. In rural China, increased funding and reimbursement for the New Rural Cooperative Medical System (NRCMS) has provided individuals with better access to inpatient care and significantly promoted the utilisation of inpatient services. The annual hospitalisation rate grew from 3.2% in 1993 to 3.4% in 2003, and then soared to 16.4% in 2015 [5]. Inpatient care was more likely to be provided by county hospitals than township hospitals. The bed utilisation ratio in county hospitals in 2016 was 84.1%, compared with 60.6% in township hospitals. The annual growth rate of inpatient admissions to county hospitals between 2010 and 2016 was 6.75%, while that of township hospitals was 0.63% [5]. This rapid growth in inpatient care has exerted a heavy financial burden on the NRCMS. Restuccia et al. (1996) has emphasised that the inappropriate admissions exist easily under the high hospital admission rates [6]. In our 2014 study, we showed that inappropriate admissions in township hospitals averaged 26.5%, and proposed that the same was true for county hospitals [7]. We hypothesised that inappropriate admissions were responsible for the sharp increases in inpatient services observed in county hospitals; providing guidance to patients and controlling inappropriate payouts would be essential to maintaining the viability of the NRCMS. Identifying inappropriate admissions is the first step in providing informed insights into this problem.

Inappropriate admissions occur worldwide and pose a challenge to health policy and management, although the underlying causes and determinants differ between countries. For technical reasons, oversight of the appropriateness of admission is relatively difficult [8,9,10]. Multiple studies have focused on assessing inappropriate admissions using expert evaluation methods [11], the opinion of the assessment team or rapid-response team [12], an intensity–severity–discharge review system with adult criteria [13,14], or an appropriateness evaluation protocol (AEP) [15,16]. AEP is considered the most effective and widely used technique, as it includes both disease severity and requirements for services as indicators of appropriate hospitalisation. A mismatch between all indicators indicates that the admission is inappropriate, while congruence on any one indicator indicates that the admission is appropriate [15,17]. By reviewing the patient’s medical records, the AEP enables evaluation of the rationality of the care. AEPs are based on medical and technical standards and not on condition severity, complications, length of stay (LOS), or expert opinion, so evaluation can be conducted by individuals who are not physicians, and can even be used in cases of undefined or incorrect diagnosis.

Compared with developed countries that successfully set up AEP standards in the 1990s, China has carried out limited research about the appropriateness of admission. Existing research has mainly focused on the appropriateness of specific services performed during the inpatient stay [18]. Some NRCMS agencies have made attempts to control the occurrence of inappropriate admissions using the LOS or hospitalisation expenses as benchmarks [19], but China lacks a specific and uniform evaluation standard.

In our previous research, we developed an AEP by expert consultation. In the present study, we modified this AEP for county hospitals, to assess the extent of inappropriate admissions in county hospitals in rural China and to identify their determinants. The new AEP can be used to compare with the foreign AEP versions.

## 2. Materials and Methods

### 2.1. Expert Consultation for AEP Criteria

We took our existing AEP for township hospitals, which comprises nine requirements for services and 20 indicators for disease severity [7], and used expert consultation to develop new AEP criteria for county hospitals. The panel consisted of 17 experts, each of whom was required to have more than five years of working experience. Of the 17 experts, three were from tertiary hospitals, six from county hospitals, six from township hospitals, and two from county NRCMS agencies. Fifteen had served on our consultant panel when we developed AEP criteria for township hospitals, and all experts were familiar with those criteria and with the status of county hospitals. The main objective was to adjust the indicators to improve the existing AEP criteria for use in county hospitals. Cronbach’s alpha coefficient of reliability was calculated to assess the expert consultation outcomes.

### 2.2. Data Sources

We conducted this research in 2016, using stratified cluster sampling. We sampled the same five counties used in our previous study (Jingshan, Songzi, Danjiangkou, and Dangyang in Hubei Province and Meixian in Shaanxi Province in midwestern China). NRCMS payments and reimbursements in the five counties are similar. Reimbursement involves patient accounts for outpatient services and a pooled fund for inpatient services. Outpatient services in county hospitals are not covered, and the reimbursement ratios for inpatients range from 60% to 80%, with a deduction of 300–500 RMB. 

Next, we used the largest county hospital in each county as the sample hospital. In the sampling calculation, the estimated inappropriate admission rate *P* is 16%, the expected error *d* = 0.1 × *P* = 1.6%, and *α* = 0.05:

N = (Z*_α_*/*d*)^2^ × *P*(1 − *P*) = (1.96/1.6%) ^2^ × 16% × (1 − 16%) = 2016.84

Cluster sampling was used to select 500 admission records from 2014 from each hospital. Considering the pertinence of AEP, we excluded hospitalisation for childbirth. We employed investigators to collect the paper medical records. In total, 2412 medical records were collected, and 2230 of these records were included in the analysis.

The socioeconomic characteristics, disease status, and admission information of patients were uploaded into Epidata version 3.2 [20]. Diseases were categorised using the International Classification of Diseases, 10th revision (ICD-10). It is worth noting that admitted severity, which we collected from medical records home page, was a specific item that came from the doctor’s subjective judgment, thus is very different from the AEP criteria indicator.

### 2.3. Appropriateness Evaluation

We screened 2230 admission records using the improved AEP. We first extracted the values of all indicators from each record manually. Then, we ran a computer algorithm against the values from the medical records, and identified inappropriate admissions if the values did not fit any AEP criteria. 

### 2.4. Statistical Analysis

The characteristics of patients inappropriately admitted to county hospitals were analysed by *t*-test and Pearson’s chi-squared test using IBM SPSS 22.0 (Armonk, NY, USA). Because the five sample counties differed in social customs, geographic location, and county hospital capacity, all of which may have contributed to the inappropriateness of admissions, the data we obtained indicated a hierarchical structure at the county level. A two-level logistic regression model in MLwiN version 2.30 was used (University of Bristol, Bristol, UK) [21]. The medical record and county were assigned to levels 1 and 2, respectively. The regression model is displayed in Equation (1).

(1) $$\begin{array}{l} \mathrm{Logit}\left(\pi_{\mathrm{ij}}\right)=\beta_{0j}\mathrm{cons}+\beta_{1}{\mathrm{Gender}}_{\mathrm{ij}}+\beta_{2}{\mathrm{Age}}_{\mathrm{ij}}+\beta_{3}{\mathrm{Department}}_{\mathrm{ij}}+\beta_{4}{\mathrm{Severity}}_{\mathrm{ij}}+\beta_{5}{\mathrm{Disease}}_{\mathrm{ij}}\\ \;+\beta_{6}{\mathrm{LOS}}_{\mathrm{ij}}+\beta_{7}{\mathrm{Complication}}_{\mathrm{ij}}+{\mathrm{More}}_{\mathrm{ij}}\\ \beta_{0j}=\beta_{0}+u_{0j} \end{array}$$

where LOS stands for length of stay, *β_i_* refers to the fixed effects parameter, and *u**_0j_*** refers to the random effects of level 2.

### 2.5. Research Ethics

The study protocol conformed to the guidelines of the Ethics Committee of Tongji Medical College of Huazhong University of Science and Technology. Patient information was anonymised and deidentified prior to the analysis.

## 3. Results

### 3.1. AEP for County Hospitals

Table 1 displays the modified AEP criteria for county hospitals. Cronbach’s alpha coefficients for the three rounds of expert consultation were 0.692, 0.842, and 0.833. The new AEP comprises nine indicators of service requirements, such as surgery needs, and 21 indicators of disease severity, such as fever (over 38 °C) persisting longer than five days. We modified the AEP criteria for township hospitals as follows [7]: Indicators A1.2 (invasive diagnostics of central skeletal muscle tissue) and B21 (burns sustained in specific areas) were added; indicator A1.1 (required local or general anaesthesia) was removed; and indicators A1.1 and B19 (infection in 50% of the lung) were modified. 

### 3.2. Characteristics of Patients Inappropriately Admitted to County Hospitals

Patient characteristics and causes of hospitalisation are shown in Table 2. The overall prevalence of inappropriate admissions in the five counties was 15.2%, and varied significantly between counties (χ^2^ = 23.9, *p* < 0.001). Danjiangkou had the highest rate of inappropriate admissions (22.5%), while Jingshan had the lowest rate (11.8%). No difference was observed in the appropriateness of admission between sexes. Inappropriate admissions were mainly found for patients aged <20 and >59 years. The inappropriate admission rate was higher for patients with complications and LOS < 8 days. Inappropriate admission was more likely in internal medicine and paediatric patients, and the rate was higher among patients with a lower severity of symptoms. In terms of the cause of hospitalisation, the inappropriate admission rate was highest for skeletal and muscular diseases (22.6%), followed by rates for respiratory and circulatory diseases (approximately 18.0%). The lowest rates of inappropriate admissions were observed in cases of injury and poisoning (7.0%).

### 3.3. Determinants of Inappropriate Admissions in County Hospitals

The level 2 variance of the null model did not show statistical significance (*p* = 0.227), so inappropriate admissions did not appear to be aggregated at the county level. We then introduced patient characteristics and causes of hospitalisation as explanatory variables into the logistic regression model with forward method (Table 3).

The two-level logistic regression analysis showed that age, treating department, cause of hospitalisation, severity of symptoms at admission, complications, and LOS were determinants of inappropriate admission in county hospitals. Inappropriate admissions were higher in younger patients. Taking admissions in the internal medicine department as a reference, the odds ratio (OR) of inappropriate admission incidence in the paediatric department was 2.124, while the OR in the surgery department was only 0.326. A high severity of symptoms at admission was correlated with a lower incidence of inappropriate admission, which occurred mainly among patients with mild symptoms. Inappropriate admission was more frequent for respiratory, circulatory, skeletal, and muscular causes. In addition, patients with complications were less likely to be inappropriately admitted (OR: 0.723). The LOS was another important determinant, and patients with a shorter hospital stay were more likely to be inappropriately admitted (OR: 0.965).

## 4. Discussion

### 4.1. Application of the AEP Criteria for County Hospitals

The AEP we developed (two categories, 30 indicators) is more specific and comprehensive than AEPs developed in the US (two categories, 16 indicators) [22], the EU (two categories, 15 indicators) [23], and The Netherlands (one category, 15 indicators) [24]. The differences may be attributed to characteristics of hospital levels and management systems [25]. The county hospital in rural China is the primary general hospital in the country, and services a wide range of individuals compared to the specialist hospitals. The medical treatments and resources offered by county hospitals in China are limited, meanwhile there is no restriction or general practitioners for residents’ choice of treatment in China, therefore AEP criteria for these hospitals should include multiple indicators to control inappropriate admission.

Our modification of the AEP for township hospitals focuses on more specific causes of hospitalisation and more variables in the analysis because of the diverse outpatient services required in county hospitals. In this setting, the AEP mainly covers internal medicine, surgery, and paediatrics, accounting for the needs of approximately 90% of the patients. Admission for childbirth was appropriate and did not require evaluation, so we excluded obstetrics cases. Moreover, this AEP can be adapted for various types of medical institutions, service delivery levels, and geographical areas.

We should note that this AEP incorporates the clinical perspective regardless of social and ethical factors [26], inpatient care may be considered more convenient and humanistic than outpatient care for those who are elderly or reside in remote areas or empty nesters.

### 4.2. Inappropriate Admission in County Hospitals in Rural China

We found an inappropriate admission rate of 15.2% in the county hospital records we surveyed. This number is higher than the 9.6% reported for nursing home residents in The Netherlands (1999) [27], the 11.0% in Portsmouth (1995) [20] and 9.1% (2000) [28] reported for geriatric units in the UK. Higher inappropriate admission rates were reported by Mytton et al. (2012) (20.6–32.0%) and McDonagh et al. (20.0–30.0%) [29] in London, and the differences may reflect the ages of the cohorts sampled or the healthcare protocols involved. 

We found that Danjiangkou County was a region with higher rates of inappropriate admission in both township (41.18%) and county (22.5%) hospitals. Inappropriate admission is associated with the design of the NRCMS reimbursement program [7], which determines the choice of medical service directly. The NRCMS allows patients in China to choose whether they want inpatient or outpatient services. Patients compare the reimbursement scale, convenience, and comfort of outpatient and inpatient services may, in some cases, choose to be admitted [30]. We did not find stark differences in inappropriate admissions between counties, perhaps because the number of counties sampled was small, while the number of patients was large [31].

### 4.3. Characteristics of Patients Hospitalised Inappropriately

We found that patient characteristics, excluding sex, influenced inappropriate admissions. The elderly had a high rate of inappropriate admissions (14.3%), although it was lower than the rate in township hospitals (30.0%). Two systematic reviews have also found a high rate of inappropriate admissions among the elderly [14,29]. We found a high incidence of inappropriate admissions among patients aged <20 years (19.3%), which was lower than the 26.0% reported for children with influenza-like symptoms in Italy, perhaps because we included all causes of hospitalisation [32]. Inappropriate admission was more frequent in the internal medicine and paediatric departments than in the surgery department, probably because surgery is an original evaluation index in the AEP, reflecting an urgent and viable need [33]. When stratifying the causes of hospitalisation, we found that patients with conditions related to the respiratory, circulatory, and skeletal and muscular systems were more likely to be inappropriately admitted, unlike patients with urinary diseases. This finding can be related to the county hospital’s capacity for diagnosis and treatment of outpatient services. Diseases of the urinary system cannot be controlled well on an outpatient basis, resulting in a high probability of appropriate hospitalisation. In contrast, respiratory, cardiovascular, and endocrine diseases can be treated and controlled well without hospitalisation, but the boundary between outpatient and inpatient departments in these cases is not clear [34], leading to inappropriate admission. Children who are inappropriately admitted are mainly diagnosed with influenza and paediatric bronchial pneumonia [32,35]; these diseases have a high incidence and a longer duration (typically 5–7 days) of symptoms. Drug treatment and adequate rest are important in recovering from these diseases, but patients may perceive hospitalisation as superior to treatment in village clinics or outpatient care [36]. Thus, physicians may agree to hospital admission at the patient’s request [37], even though the severity of the disease and the required medical services do not meet the admission criteria. 

We found a higher rate of inappropriate admissions among patients without complications, as complications imply serious illness and a more urgent requirement for treatment. The inappropriate admission rate decreased with an increase in the LOS, and was also associated with the services required. More services being needed results in longer LOS and a lower inappropriate admission rate. 

### 4.4. Redesigning the Health Delivery System to Reduce Inappropriate Admissions

The rural healthcare delivery system should specifically focus on inappropriate admissions. Establishing a monitoring mechanism for inappropriate admission is a primary and key measure, and our AEP for county hospitals can serve as a monitoring tool. Paediatric and elderly patients and those with respiratory, circulatory, and skeletal or muscular conditions warrant special attention. In addition, the motivating mechanism of county hospitals should be changed from the volume of services provided to the appropriateness of care [38]. We recommend that new mechanisms, such as a global budget for multilevel institutions [39] and integrated delivery of medicine and nursing services to the elderly, be implemented to guide patients seeking primary healthcare. Hundreds of thousands of patients may avoid hospital admission if cared for appropriately by community services [14].

## 5. Conclusions

We found that, in rural China, the inappropriate admission rate in county hospitals is very high, although it is lower than the rate in township hospitals. Age, disease severity, and disease type affect the appropriateness of admission. An advanced AEP is helpful in establishing an admission oversight system in county hospitals.

## Figures and Tables

**Table 1:** Appropriateness evaluation protocol (AEP) criteria for county hospitals.

**A. Needed medical service**
A1. Need follow-up treatment within 24 h: (1) instruments or other facilities that are only available for hospitalised patients (angiography, visceral biopsy, *cardiac catheterisation intervention*) and/or (2) *invasive diagnostic of central skeletal muscle meat (lumbar puncture, cisterna puncture, ventricular puncture, encephalography)*
A2. Treatment with varying dosages or drugs on a regular basis under direct medical supervision
A3. Calculation of intake and output volume
A4. Operation to be conducted on the following day in the operating room, detailed pre-operative consultation or evaluation on the day of admission
A5. Main surgical incision and drainage nursing
A6. Quarantined patients
A7. Bedside electrocardiogram (ECG) monitoring or testing vital signs at least every 2 h
A8. Stopping (at least once every 8 h) or continuing oxygen inhalation
A9. Referral of post-operative recovery
**B. Severity of illness**
B1. Continuous fever > 38.0 °C for more than 5 days
B2. Acute confusion (coma or adiaphoria)
B3. Severe anomaly in electrolyte or blood and vigour, showing the following situations: (1) Na < 123 mEq/L or >156 mEq/L; (2) K < 2.5 mEqt/L or >6.0 mEq/L; (3) HCO_3_ < 20 mEq/L or >36 mEq/L; and (4) arterial blood pH < 7.30 or >7.45
B4. Loss of sight or hearing for 48 h
B5. Loss of activity in any part of the body for 48 h
B6. Excretion disorder or absence of intestinal peristalsis in the past 24 h
B7. Active bleeding
B8. Needing blood transfusion because of bleeding
B9. Mental disorders caused by non-alcohol dependence
B10. Viscera removal or surgical wound dehiscence
B11. Pulse less than 50 or greater than 140 beats per minute
B12. *Abnormal blood pressure: systolic blood pressure < 90 mmHg or >200 mmHg and/or diastolic blood pressure < 60 mmHg or >120 mmHg*
B13. Ventricular fibrillation or acute myocardial ischemia shown by electrocardiogram (ECG) report or course log
B14. Acute haematopathy, severe medium-sized leukopaenia, thrombocytopaenia, leukocytosis, erythrocytosis, thrombocytosis or haemolysis-resulted symptoms
B15. Progressive acute neurological disorders
B16. Soft tissue injuries affecting basic self-care
B17. Acute myocardial infarction or cerebrovascular accident (stroke)
B18. Spinal cord lesions
B19. Lung infection above 50% or leafy lesions according to X-ray examination
B20. Hyperemesis or acute pain caused by acute or chronic diseases
B21. *Burns occurred in specific areas*

Italics indicate modifications from the AEP for township hospitals.

**Table 2:** Distribution of cases and appropriateness of admission (*n* = 2230).

Variable	All (Column %)	Appropriateness of Admission		*p*-Value
		Yes Number (Line %)	No Number (Line %)	
All	2230	1892 (84.8)	338 (15.2)	
County				<0.001 *
Songzi	410 (18.4)	355 (86.6)	55 (13.4)	
Dangyang	395 (17.7)	332 (84.1)	63 (15.9)	
Jingshan	527 (23.6)	465 (88.2)	62 (11.8)	
Danjiangkou	404 (18.1)	313 (77.5)	91 (22.5)	
Meixian	494 (22.2)	427 (86.4)	67 (13.6)	
Sex				0.851 *
Male	1111 (49.8)	941 (49.7)	170 (50.3)	
Female	1119 (50.2)	951 (50.3)	168 (49.7)	
Age, years				0.042 *
Less than 20	466 (20.9)	376 (80.7)	90 (19.3)	
20–39	225 (10.1)	196 (87.1)	29 (12.9)	
40–59	811 (36.4)	696 (85.8)	115 (14.2)	
More than 59	728 (32.6)	624 (85.7)	104 (14.3)	
Mean (SD)	44.7 (24.7)	45.2 (24.3)	42.1 (26.7)	<0.001 **
Treating department				<0.001 *
Internal medicine	867 (38.9)	712 (82.1)	155 (17.9)	
Surgery	558 (25.0)	512 (91.8)	46 (8.2)	
Paediatrics	319 (14.3)	246 (77.1)	73 (22.9)	
Others	486 (21.8)	422 (86.8)	64 (13.2)	
Admitted severity				<0.001 *
Serious	622 (27.9)	601 (96.6)	21 (3.7)	
Urgent	923 (41.4)	759 (82.2)	164 (17.8)	
General	685 (30.7)	532 (77.7)	153 (22.3)	
Causes of hospitalisation				0.001 *
Urinary disease	479 (21.5)	412 (86.0)	67 (14.0)	
Respiratory disease	504 (22.6)	411 (81.5)	93 (18.5)	
Circulatory disease	377 (16.9)	311 (82.5)	66 (17.5)	
Digestive disease	210 (9.4)	182 (86.7)	28 (13.3)	
Injury and poisoning	186 (8.3)	173 (93.0)	13 (7.0)	
Symptoms and signs	249 (11.2)	223 (89.6)	26 (10.4)	
Skeletal and muscular disease	93 (4.2)	72 (77.4)	21 (22.6)	
Others	132 (5.9)	108 (81.8)	24 (18.2)	
Complications				0.006 *
Yes	957 (42.9)	835 (87.3)	122 (12.7)	
No	1273 (57.1)	1057 (83.0)	216 (17.0)	
Length of stay				<0.001 *
Less than 8 days	1067 (47.8)	870 (81.5)	197 (18.5)	
8–14 days	877 (39.3)	767 (87.5)	110 (12.5)	
More than 14 days	286 (12.8)	255 (89.2)	31 (10.8)	
Mean (SD)	8.9 (6.1)	9.11 (6.2)	7.87 (5.1)	0.002 **

* Pearson’s chi-squared test; ** *t*-test.

**Table 3:** Two-level logistic regression analysis of inappropriate admissions (*n* = 2230).

Variable	Estimated Value	Standard Error	*F*	*p*-Value	Adjusted OR
Fixed factors					
Constant	−1.083	0.336	10.387	0.001	0.339
Sex (male)					
Female	−0.115	0.127	0.822	0.365	0.891
Age (year)	0.011	0.004	7.017	0.008	0.987
Department (internal medicine)					
Surgery	−1.121	0.214	27.422	0.000	0.326
Paediatrics	0.754	0.283	7.081	0.008	2.124
Other	−0.658	0.185	12.634	0.000	0.518
Admitted severity (general)					
Urgent	−0.290	0.131	4.919	0.027	0.748
Serious	−2.475	0.291	72.225	0.000	0.084
Causes of hospitalisation (urinary disease)					
Respiratory disease	0.407	0.232	3.073	0.008	1.503
Circulatory disease	0.412	0.221	3.469	0.043	1.510
Digestive disease	0.035	0.259	0.018	0.892	1.036
Injury and poisoning	−0.324	0.360	0.002	0.009	0.628
Symptoms and signs	−0.055	0.275	0.040	0.008	0.946
Skeletal and muscular disease	0.898	0.315	8.110	0.004	2.455
Other	0.426	0.301	2.007	0.157	1.532
Complication (no)					
yes	−0.325	0.143	5.177	0.023	0.723
Length of stay (day)	−0.036	0.013	8.220	0.004	0.965
Random factors					
County variance	0.007	0.001	9.532	0.227	
Record scale parameter	1.000	0.000	–	–	

Age and length of stay were used as measurement data; severity of symptoms was ranked in order. OR, odds ratio.

## Abbreviations

AEP	Appropriateness evaluation protocol
LOS	Length of stay
NRCMS	New Rural Cooperative Medical System
